# Distribution of *pfmdr1* and *pfcrt* chloroquine drug resistance alleles in north-western Nigeria

**Published:** 2017-08-01

**Authors:** Ruqayyah H. Muhammad, Ishaya H. Nock, Iliya S. Ndams, Jonathan B. George, Yusuf Deeni

**Affiliations:** 1 Federal University Dutse, PMB 7156 Jigawa state, Nigeria; 2 Ahmadu Bello University, Zaria, Nigeria; 3 University of Abertay, Bell Street Dundee DD1 1HG, UK

## Abstract

**Background:**

In Nigeria, decline in the sensitivity of *Plasmodium falciparum* to Artemisinin Combination Therapy (ACT) has prompted the unofficial use of chloroquine (CQ) for self-medication. This study was designed to determine the prevalence and distribution of CQ resistant/susceptible alleles of CQ resistance transporter (*Pfcrt*) and *P. falciparum* multidrug resistance gene 1 (*Pfmdr1*) in view of the possible re-introduction of CQ for malaria treatment.

**Materials and methods:**

Four hundred and sixty six (466) *P. falciparum* positive samples were randomly collected from five states of northwest Nigeria. The samples were amplified using RT- PCR at codon 76 for *Pfcrt* and codon 86 for *Pfmdr1*. Data was analysed using chi-square, odds ratios and paired t-tests.

**Results:**

Drug susceptible alleles (N86) were most prevalent in the study population (47.9%; 223/466), followed by the drug resistance alleles 86Y (28.3%; 132/466), followed by the drug susceptible alleles K76 (17.4%; 81/466), the resistant alleles 76T (12.4%; 58/466) and finally the mixed infection mutation K76T (3.6%; 17/466). Differences between the distributions of the *Pfmdr1* and *Pfcrt* alleles were significant (P<0.05). There were significant differences (P<0.05) between N86 and 86Y alleles, but no significant differences between K76 and 76T alleles, including the prevalence of the various alleles across the different age groups.

**Conclusion:**

The results of this study suggest the possibility of (re)introducing CQ for malaria treatment in north-western Nigeria and provide insight in the genetic background of *P. falciparum* in the study area.

## 1 Introduction

Malaria remains a major health problem in Sub-Saharan Africa [1] and the world’s most important parasitic infection, ranking among the major health and developmental challenges for developing countries [2,3]. Although there are encouraging reports that malaria morbidity and mortality are declining, there are globally still an estimated 207 million cases and 627 thousand deaths every year. The entire Nigerian population of >170 million is at risk of malaria, which is responsible for about 60% and 30% of outpatient visits and hospital admissions, respectively [4]. Emergence and spreading of clones of *P. falciparum* resistant to the most commonly available antimalarial drugs hinders effective control of the disease. Chloroquine (CQ) was the first antimalarial to be widely used in endemic countries, including Nigeria. Resistance to it was first documented in Thailand in the late 1950’s, and spread to Africa in the 1970s. It surfaced in Nigeria in the early 1980’s and continued to spread until 2005 when CQ was withdrawn. The Nigerian government subsequently changed its first line drug to the more expensive Artemisinin based Combination Therapy (ACT) and recommended that all fevers be treated presumptively with ACTs where confirmation could not be obtained [5]. However, since the detection of *P. falciparum* resistant to ACT in western Cambodia, malaria control and elimination efforts have become seriously threatened [6].

In different parts of Nigeria, *P. falciparum* resistance to CQ has prompted studies in the last decade [7-10]. While high resistance to CQ was reported in south-eastern Nigeria [8], most strains of *P. falciparum* were found to be fully sensitive to CQ in north-eastern Nigeria [7]. Erah *et al.* [11] showed that CQ is still effective in the treatment of uncomplicated malaria in Delta state.

Chloroquine resistant *P. falciparum* malaria is caused by mutations in two genes, the *P. falciparum* CQ resistance transporter (*Pfcrt*) and multidrug resistance transporter-1 (*Pfmdr1*), which are both located on the food vacuole of the parasite. While wild-type *Pfmdr1* is thought to transport and accumulate CQ into the parasite food vacuole, mutations N86Y, S1034C, N1042D, and D1246Y abolish this transport, leading to reduced CQ sensitivity [12]. On the other hand, the CQ transporter *Pfcrt* is a stronger predictor of CQ resistance than *Pfmdr1*. Mutations on the *Pfcrt* K76T is directly linked with both *in vitro* and clinical resistance and is thus used as a biomarker of CQ resistance [13]. As polymorphisms of the mutations have also been linked to resistance or sensitivity to other antimalarial drugs, the withdrawal of drugs may have resulted in the re-emergence of sensitive genes as has been documented following CQ withdrawal. In Malawi, parasites carrying the CQ sensitive *pfcrt* 76, with 100% clinical efficacy, was reported just eight years after abandoning its use [14]. Two studies in Tanzania reported restoration of CQ-sensitive *pfcrt* 76 from 17.1% to 50.7% in five years [15] and from 48% to 89.6% in seven years [16]. In Kenya the restoration was slower than in Malawi and Tanzania, rising from 5% to 40% in 13 years [17].

This present study investigated the prevalence and distribution of *pfcrt* and *pfmdr1* susceptible and resistance alleles in clinical isolates from five states of north-western Nigeria, for possible re-introduction of CQ for malaria treatment in that part of the country.

## 2 Materials and methods

### 2.1 Study area

**Figure 1:**
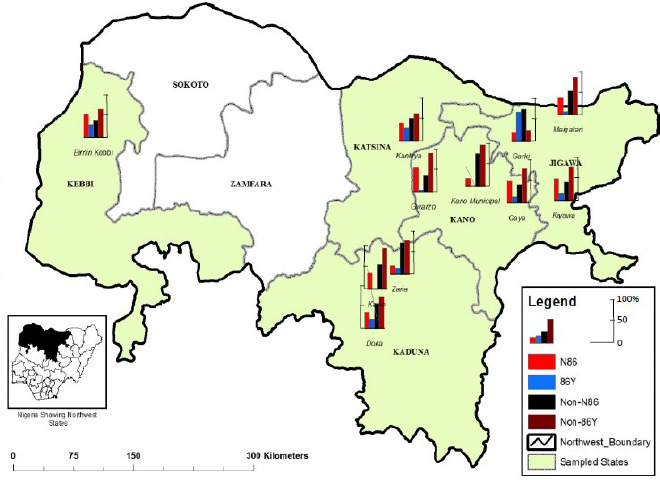
Distribution of *P. falciparum pfmdr1* alleles in five states of northwest Nigeria. Map source: Cartography Laboratory, Bayero University, Kano. For data see also Supplementary file 1.

The study was conducted in randomly selected States of north-western Nigeria (Figure 1). Mosquito breeding sites abound in the region due to farming, industrial and other human activities contributing to larval growth and development [18]. Malaria is meso- to hyper-endemic in the States and is seasonally transmitted, with the main peak of transmission from early June to late August and a minor peak from early October to mid November. These transmission periods correspond with rainfall patterns and resulting high populations of mosquitoes and intense *P. falciparum* transmission [19].

### 2.2 Blood collection and sample handling

For *P. falciparum* screening, 2-5 ml of venous blood was collected from hospital patients by clinicians or medical laboratory scientists after obtaining the patient’s consent and demographics. Blood was stored in sample bottles containing EDTA as anti-coagulant. Both thin and thick smears of each sample were Giemsa stained for microscopic analysis. Four hundred and sixty six (466) *P. falciparum*-positive samples were blotted on Whatman filter paper (24 cm) in quadruplets. These were allowed to dry and stored in a separate clean envelope for molecular analysis. A sterile paper punch (6 mm) was used to cut out the dry blood spots (DBS). DNA was extracted from the DBS using a modified Qiagen protocol (QIAamp) DNA blood kit (Qiagen, Hilden, Germany).

### 2.3 Real time polymerase chain reaction (RT-PCR)

RT-PCR was used to determine the susceptible and resistant alleles of *Pfcrt* at codon K76T and *Pfmdr1* at codon N86Y in positive samples using specific primers adopted from the work of Ojurongbe *et al.* [20], and designed by Sysmex UK Ltd. The primers and probes used are shown in Table 1.

**Table 1:** Primers, probes and melting temperatures for *P. falciparum* chloroquine resistance transporter (*pfcrt*) and *P. falciparum* multidrug resistance gene 1 *(pfmdr1).*

	Primers and probes used	Melting temperatures (°C)
*Pfcrt*	F: 5’-CTTGTCTTGGTAAATGTGCTCA-3’	Wild: 65.3 ± 0.4
	R:5’-GTTACCAATTTTGTTTAAAGTTCT-3’	Mutant: 46.5 ± 0.2
	SensorProbe 76:	
	SPTGTGTAATTGAAACAATTTTTGCTAA-3	
*Pfmdr1*	F:5’-TGTATTATCAGGAGGAACATTACC-3’	Wild: 51.8 ± 0.3
	R:5’- ACCACCAAACATAAATTAACGGA-3’	Mutant: 56.5 ± 0.2
	SensorProbe86:	
	ATTAATATCATCATAAATACATG 51	
	AnchorProbe86:	
	TCTTTAATATTACACCAAACACAGATAT	

The samples were analysed for *pfcrt* alleles at codon 76 (lysine to threonine). The sensor probe labelled with fluorescein at the 3' end was designed to be perfectly complementary to the mutation site. An amplification primer iLC labelled with Cy5 on the third base from the 3' end was used as a reverse primer, which is extended during amplification. During fluorescence Resonance Energy Transfer (FRET), fluorescein, which is excited by the light source of the Rotor Gene instrument transfers its energy to the Cy5 incorporated into the PCR product working as anchor probe [21,22]. A specific melting temperature is then obtained for each genotype: a sensor probe spanning one mismatch could still hybridise to the target sequence but will melt off at lower temperature than a sensor probe with a perfect match [20].

The samples were also analysed for the *pfmdr1* alleles at codon 86 (asparagine to tyrosine). *Pfmdr1* allele’s hybridisation probes consisted of two different oligonucleotides that bind to an internal sequence amplified by forward and reverse primers. The sensor probe, labelled at the 3' end with FAM, is designed to match the mutation sites. The anchor probe, labelled at the 5' end with Cy5 and phosphorylated at the 3' end, to prevent extension by Taq polymerase, is designed to conserve sequences adjacent to the mutation sites. Both probes, localised on the same DNA strand, could hybridise in a head to tail arrangement, bringing the two fluorescent dyes into close proximity. During Fluorescence Resonance Energy Transfer (FRET), FAM was excited by the light source of the Rotor Gene instrument. The excitation energy was transferred to the acceptor fluorophore, Cy5, and the emitted fluorescence was measured continuously on the Rotor Gene channel during the melting phase.

### 2.4 Ethical clearance

Scientific and Ethical permit/clearance was obtained from the Ministries of Health/Hospital Management Board (MOH/HMB) of Kano, Kaduna, Katsina, Kebbi, and Jigawa States before commencing the research. Written/informed consent was obtained from patients prior to recruitment into the study. Consent from children was provided by the parents/guardians.

## 3 Results

A total of four hundred and sixty six (466) *P. falciparum*-positive samples were amplified at codon 76 for the *pfcrt* gene and at codon 86 for the *pfmdr1*gene. Drug susceptible alleles (N86) were more prevalent (47.9%; 223/466) in the study population, followed by the drug resistance alleles 86Y(28.3%; 132/466), followed by the drug susceptible K76 (17.4%; 81/466), the resistant 76T (12.4%; 58/466) and finally the mixed infection mutation K76T(3.6%; 17/466). There was a significant difference (P≤0.05) between the geographical distributions of the *Pfmdr1* and the *Pfcrt* alleles (Table 2).

**Table 2:** Geographical distribution of *pfcrt* and *pfmdr1* alleles in the study population.

State	# Examined	*Pfcrt*			*Pfmdr1*	
		K76	76T	K76T	N86	86Y
Kaduna	53	17 (32.1)	10 (18.9)	3 (5.67)	18 (34.0)	10 (18.9)
Jigawa	43	8 (18.6)	11 (25.6)	5 (11.6)	17 (39.5)	14 (32.6)
Kano	53	13 (24.5)	9 (17.0)	1 (1.9)	28 (52.8)	6 (11.3)
Kebbi	135	10 (7.4)	7 (5.2)	2 (1.5)	78 (57.8)	42 (31.1)
Katsina	182	33 (18.1)	21 (11.5)	6 (3.3)	82 (45.1)	60 (33.0)
Total	466	385 (82.6)	58 (12.4)	17 (3.6)	223 (47.9)	132 (28.3)
	Chi-square	19.3	16.5	6.3	11.7	12.7
	df	4	4	4	4	4
	p value	0.001	0.002	0.181ns	0.020	0.013

Based on gender both K76 and 76T were more frequently found in females (18.8%; 52/276, and 12.4%; 58/276, respectively). The mixed mutation infection K76T was more common in males (4.2%; 8/190). The results also showed that both N86 and 86Y were more commonly found in males (47.9 %; 91/190 and 32.1%; 61/190, respectively). Overall there was no significant difference between the sexes as well as between the alleles (Table 3).

**Table 3:** Distribution of *pfcrt* and *pfmdr1* alleles according to gender.

Gender	# Examined	*Pfcrt*			*Pfmdr1*	
		K76	76T	K76T	N86	86Y
Male	190	29 (15.3)	22 (11.6)	8 (4.2)	91 (47.9)	61 (32.1)
Female	276	52 (18.8)	36 (13.0)	9 (3.3)	132 (47.8)	71 (25.7)
Total	466	81 (17.4)	58 (12.4)	17 (3.6)	223 (47.9)	132 (28.3)
	Chi-square	1.00	0.22	0.29	0.000	2.26
	df	1	1	1	1	1
	p value	0.32ns	0.64ns	0.59ns	0.99ns	0.13ns
	Odds ratio	0.78	0.87	1.30	1.00	1.37
	95% CI	0.47-1.27	0.49-1.54	0.49-3.44	0.69-1.45	0.91-2.05

Age wise, K76 was found more often in the age group 6-15 yrs (21.8%; 52/78), 76T was highest in the age group 26-40 yrs (17.0%; 17/100) while the mixed mutation infection (K76T) was higher in the 16-25 yrs age group 6.9% (6/87). Also N86 was higher among the age group of > 40 yrs of age (52.8%; 28/53) while 86Y was higher among the age group 1-5 yrs (31.1%; 46/148). But like for gender, there was no significant difference between the ages the alleles (Table 4).

**Table 4:** Distribution of *pfcrt* and *pfmdr1* alleles according to age.

Age (yrs)	# Examined	*Pfcrt*			*Pfmdr1*	
		K76	76T	K76T	N86	86Y
1-5	148	25 (16.9)	16 (10.8)	4 (2.7)	70 (47.3)	46 (31.1)
6-15	78	17 (21.8)	11 (14.1)	3 (3.8)	36 (46.2)	22 (28.2)
16-25	87	17 (19.5)	13 (14.9)	6 (6.9)	40 (46.0)	24 (27.6)
26-40	100	13 (13.0)	17 (17.0)	3 (3.0)	49 (49.0)	24 (24.0)
>40	53	9 (17.0)	1 (1.9)	1 (1.9)	28 (52.8)	16 (30.2)
Total	466	81 (17.4)	58 (12.4)	17 (3.6)	223 (47.9)	132 (28.3)
	Chi square	2.708	8.383	3.584	0.81	1.590
	df	4	4	4	4	4
	p value	0.61ns	0.08ns	0.47ns	0.94ns	0.81ns

Both the drug resistance and susceptible alleles of the *pfmdr1* and *pfcrt* genes were widely distributed in the study area. *Pfmdr1* drug susceptible alleles (N86) were most prevalent in Kebbi State (57.8%; 78/135) and least so in Kaduna State (34.0%; 18/53). The *pfmdr1*drug resistance alleles were most commonly found in Katsina State (33.0%; 60/182) and least so in Kano State (11.3%; 6/53) (Table 2 and Figure 1; data shown in Supplementary file 1).

**Figure 2:**
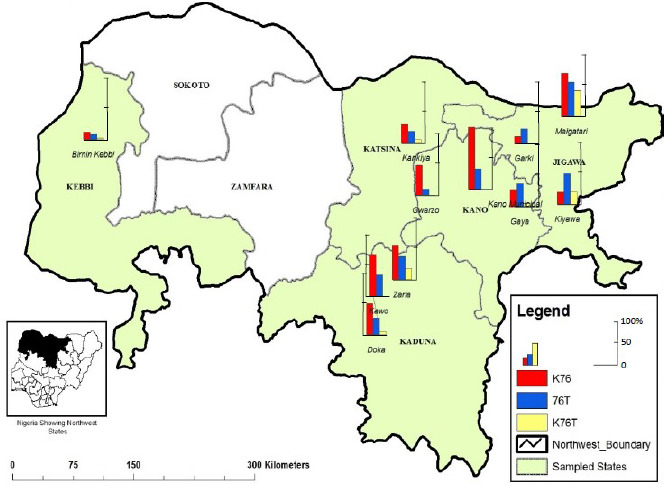
Distribution of *P. falciparum pfcrt* alleles in five states of northwest Nigeria. Map source: Cartography Laboratory, Bayero University Kano. For data see also Supplementary file 1.

*Pfcrt* drug susceptible alleles K76 were most prevalent in Kaduna State (32.1%; 17/53), and least so in Kebbi State (7.4%; 10/135). The *pfcr*t drug resistance alleles 76T were more common in Jigawa State (25.6%; 11/43) and least common in Kebbi State (5.2%; 7/135) (Table 2). K76T mixed infection mutation were most prevalent in the following Jigawa State (11.6%; 5/43) and again least so in Kebbi State (1.5%; 2/135) (Table 2 and Figure 2).

## 4 Discussion

The high prevalence of malaria in most parts of Nigeria, coupled with the sole reliance on ACT treatment and the withdrawal of CQ since 2005, could have resulted in the selection and propagation of ACT resistance and re-emergence of CQ susceptible alleles. This study also showed that the CQ susceptible alleles of the two genes *pfmdr1*and *pfcrt* were prevalent (47.9% and 17.4%, respectively) in the study population, which could be the result of withdrawal of CQ for malaria treatment or drug pressure that is mounting on artemether-lumefantrine (AL). It further suggests that CQ can be used for malaria treatment since *pfcrt*, K76, and *pfmdr*1, N86, do not confer resistance to CQ [23,24].

The occurrence of mutant parasites with *pfcrt* T76 (28.3%) and *pfmdr*1Y86 (12.4%) is a threat to some of the ACTs in use, suggesting drug pressure. This was shown in an *in vitro* study conducted in southeast Nigeria, which demonstrated an association between the T76 mutation and decreased susceptibility to artemether [25]. In addition, an increasing trend for K76 will create a future problem for ACT use because it has been seen in recrudescent samples after AL use [26]. The mixed mutation, *pfcrt* K76T, is directly linked with both *in vitro* and clinical resistance and is thus used as a biomarker of CQ resistance [13]. Another report, from Ibadan, Nigeria, suggested an association between the *pfcrt* T76 and *pfmdr*1 Y86 alleles in CQ-resistant isolates [27].

The fact that the drug resistance alleles 86Y were higher in males (32.1%) than in females (25.7%) implies that males stand a greater chance of developing drug resistance than females. Also the higher occurrences of the drug susceptible alleles N86 in males suggest that CQ may be used to cure the disease in that gender. Moreover the higher presence of *pfcrt* K76T mixed infection alleles in males (4.2%) further buttressed the fact above that males are more likely to develop drug resistance because the presence of K76T mutation is pre-condition for the parasite to develop multidrug resistance property against CQ [28].

Both *pfmdr* drug susceptible alleles N86 and 86Y were prevalent across the various age groups with no clear demarcation. This may be caused by the withdrawal of CQ for malaria treatment or it could be due to a mounting pressure on AL or both. Also the *pfcrt* drug susceptible alleles K76 and drug resistant alleles 76T and K76T mixed infections alleles were higher among different age groups, suggesting similar pressure on the drugs. These results further suggest possible drug pressure among the age groups that resulted in declined drug susceptibility or that the effect of CQ withdrawal for about a decade resulted in the CQ susceptibility increase. Although there are no previously documented data in the study area to support these findings, studies have shown that removal of CQ for the treatment of *P. falciparum* or the pressure from AL that has been used eventually lead to replacement of *pfmdr1* resistance genes by susceptible parasite populations [29,30]. The findings are consistent with observations from Malawi [29], Kenya [30] and Tanzania [31], where the withdrawal of CQ resulted in the rapid spread of a CQ susceptible *pfcrt* K76 population. In Malawi, recovery of the susceptible *pfcrt* K76 from <15% to 100% within 13 years of CQ withdrawal was reported by Mang’era *et al.* [29].

The high distribution level of both drug susceptible and drug resistance alleles observed in this study showed that either CQ remained widely used at the community level even 10 years after its official withdrawal or that drug pressure is mounting on AL, or both. This might have been caused by poor education of drug retailers during the change over period, or simply due high cost of ACT, that made patients to continue using the much cheaper CQ. As there is no documented record, it is difficult to determine whether the CQ-susceptible resurgence is due to back mutations in the CQ-resistant alleles or the expansion of surviving CQ-susceptible reservoir populations. However re-expansion appears to be more common in Africa, where transmission rates are higher and naturally immune individuals are more common than in southeast Asia where CQ-resistant alleles appear to have gone to fixation in many areas [29].

*Pfmdr1* drug susceptible alleles N86 were more prevalent Kebbi state (57.8%), followed by Kano state (52.8%), Katsina (45.1%), Jigawa state (39.5%) and Kaduna state (33.96%). These results showed that there has been strong selection for CQ-susceptible parasites after the nationwide replacement of CQ with AL in 2005. The return of CQ-susceptible alleles in a country like Nigeria which is endemic for malaria, can be considered as a positive development toward possible replacement of expensive AL with a safe and cheaper drug (CQ).

The lower distribution of *pfmdr1* drug resistance alleles 86Y across the states, 33.0% in Katsina, 32.56% in Jigawa, 31.1% in Kebbi, 18.9% in Kaduna and 11.32% in Kano, suggests recovery of the wild type alleles, as the same scenario was observed for *pfmdr1* N86Y, where there was a decline in the prevalence of the mutant from 46% to 28% from 2005 to 2010 and an increase in the prevalence of the wild type strains N86 from 77% to 86% in Ghana [32]. Similar observations have been made in other studies in Africa where K76T and N86Y were investigated concurrently [33]. This is also an indication of a gradual gain of stability of these genotypes in the population similar to what was observed by Nzila’s group in Kenya [17].

As the drug susceptible allele K76 was more prevalent in almost all the locations than the drug resistance allele 76T (Figure 2) this suggests either a gradual recovery of the susceptible alleles over the period since the CQ withdrawal, or it could be due partial withdrawal of CQ for *P. falciparum* treatment, or both. In similar studies like in coastal Tanzania, the prevalence of mutant alleles of *pfcrt* decreased after only 2.5 years of CQ withdrawal [34]; Malawi showed 100% recovery of the mutant alleles after a decade of non-use CQ [14].

In addition, an increasing trend for K76 will create a future problem for ACT use as it has been observed in recrudescent samples after AL use [26]. Finally, factors such as farmland activities, especially irrigation farming, might also have contributed to increased transmission and the higher prevalence of resistance alleles obtained in Jigawa and Kaduna States. Mohammed *et al* [31] suggested that factors such as differences in malaria transmission pattern and intensity may also play a role.

## 5 Conclusions and recommendation

This study concludes that *pfcrt* and *pfmdr1* resistant alleles are prevalent in the study area but the prevalence is neither influenced by gender nor by age. The study provided insight into the genetic background of *P. falciparum* parasites prevalent in north-western Nigeria. The possibility of using CQ in future as a combination therapy with other short acting drugs with different pharmacokinetic and pharmacodynamic profiles will be an additional antimalarial option. Since CQ-resistant alleles are relatively rare, the drug may be introduced as prophylaxis for malaria risk groups, such as children and pregnant women. There is also the need for continuous monitoring of the molecular markers to be able to establish a trend of drug resistance or susceptibility in this part of Nigeria.

## Supporting information

**Table 1:** Distribution of *Pfcrt* alleles in the senatorial districts of Kano State.

Senatorial Districts	Number Examined	*Pfcrt* K76		*Pfcrt* 76T		*Pfcrt* K76T	
		K76	Non-K76	76T	Non-76T	K76T	Non-K76T
N-S (Gaya)	17	5 (29.4)	12 (70.6)	1 (5.9)	16 (94.1)	0 (0.0)	17 (100.0)
N-W (Gwarzo)	31	5 (16.1)	26 (83.9)	7 (22.6)	24 (77.4)	1 (3.2)	30 (96.8)
N-C (Municipal)	5	3 (60.0)	2 (40.0)	1 (20.0)	4 (80.0)	0 (0.0)	5 (100.0)
Total	53	13 (24.53)	40 (75.47)	9 (16.98)	44 (83.02)	1 (1.89)	52 (98.11)
	Chi square	4.799		2.207		0.723	
	df	2		2		2	
	P value	0.091ns		0.332ns		0.697ns	

**Table 2:** Distribution of *Pfmdr1* alleles in the senatorial districts of Kano State.

Senatorial Districts	Number Examined	*Pfdmr*1 N86		*Pfdmr*1 86Y	
		N86	Non-N86	86Y	Non-86Y
N-S (Gaya)	17	10 (58.8)	7 (41.2)	1 (5.9)	16 (94.1)
N-W (Gwarzo)	31	17 (54.8)	14 (45.2)	5 (16.1)	26 (83.9)
N-C (Municipal)	5	1 (20.0)	4 (80.0)	0 (0.0)	5 (100.0)
Total	53	28 (52.83)	25 (47.17)	6 (11.32)	47 (88.68)
	Chi square	2.458		1.853	
	df	2		2	
	P value	0.293ns		0.396ns	

**Table 3:** Distribution of *pfcrt* alleles in the senatorial districts of Jigawa State.

Senatorial Districts	Number Examined	*Pfcrt* K76		*Pfcrt* 76T		*Pfcrt* K76T	
		K76	Non-K76	76T	Non-76T	K76T	Non-K76T
N-W (Garki)	14	1 (7.1)	13 (92.9)	2 (14.3)	12 (85.7)	0 (0.0)	14 (100.0)
N-C (Kiyawa)	17	2 (11.8)	15 (88.2)	5 (29.4)	12 (70.6)	2 (11.8)	15 (88.2)
N-E (Maigatari)	12	5 (41.7)	7 (58.3)	4 (33.3)	8 (66.7)	3 (25.0)	9 (75.0)
Total	43	8 (18.60)	35 (81.40)	11 (25.58)	32 (74.42)	5 (11.63)	38 (88.37)
	Chisquare	5.954		1.448		3.931	
	df	2		2		2	
	P value	0.051ns		0.485ns		0.140ns	

**Table 4:** Distribution of *pfmdr 1* alleles in the senatorial districts of Jigawa State.

Senatorial Districts	Number Examined	*Pfdmr*1 N86		*Pfdmr*1 86Y	
		N86	Non-N86	86Y	Non-86Y
N-W (Garki)	14	3 (21.4)	11 (78.6)	10 (71.4)	4 (28.6)
N-C (Kiyawa)	17	9 (52.9)	8 (47.1)	3 (17.6)	14 (82.4)
N-E (Maigatari)	12	5 (41.7)	7 (58.3)	1 (8.3)	11 (91.7)
Total	43	17 (39.53)	26 (60.47)	14 (32.56)	29 (67.44)
	Chisquare	3.221		14.562	
	df	2		2	
	P value	0.200ns		0.001**	

**Table 5:** Distribution of *pfcrt* alleles in the senatorial districts of Kaduna State.

Senatorial Districts	Number Examined	*Pfcrt* K76		*Pfcrt* 76T		*Pfcrt* K76T	
		K76	Non-K76	76T	Non-76T	K76T	Non-K76T
N-S (Doka)	30	9 (30.0)	21 (70.0)	5 (16.7)	25 (83.3)	1 (3.3)	29 (96.7)
N-W (Zaria)	18	6 (33.3)	12 (66.7)	4 (22.2)	14 (77.8)	2 (11.1)	16 (88.9)
N-C (Kawo)	5	2 (40.0)	3 (60.0)	1 (20.0)	4 (80.0)	0 (0.0)	5 (100.0)
Total	53	17 (32.08)	36 (67.92	10 (18.87)	43 (81.13)	3 (5.66)	50 (94.34)
	Chisquare	0.217		0.231		1.606	
	df	2		2		2	
	P value	0.897ns		0.891ns		0.448ns	

**Table 6:** Distribution of *pfmdr*1 alleles in the senatorial districts of Kaduna State.

Senatorial Districts	Number Examined	*Pfdmr1* N86		*Pfdmr1* 86Y	
		N86	Non-N86	86Y	Non-86Y
N-S (Doka)	30	12 (40.0)	18 (60.0)	7 (23.3)	23 (76.7)
N-W (Zaria)	18	4 (22.2)	14 (77.8)	3 (16.7)	15 (83.3)
N-C (Kawo)	5	2 (40.0)	3 (60.0)	0 (0.0)	5 (100.0)
Total	53	18 (33.96)	35 (66.04)	10 (18.87)	43 (81.13)
	Chisquare	1.675		1.611	
	df	2		2	
	P value	0.433ns		0.447ns	
